# To the Editor of the Journal of Psychological Medicine

**Published:** 1855-01-01

**Authors:** J. Laycock


### TO THE EDITOR OF THE JOURNAL OF PSYCHOLOGICAL MEDICINE.

My dear Sir,—In the current number of your Journal (October, 1854) there is an article on Non-mechanical Restraint in the Treatment of the Insane, in which I am classified with those "Medical Superintendents of Asylums who do not use restraint, but who give no opinion on the abstract question." Will you permit me to say that this is not the exact position I wish or ought to occupy, for I direct mechanical restraint (suitable to the case) to be used whenever, after a careful consideration of all the circumstances, it appears to me the best for the patient. *Practically*, I think I differ little, if at all, from the great majority of medical superintendents in the *abstract* opinion; for seclusion, immersion in the cold bath, the cold shower bath, and various other methods used to secure a control of the actions of the insane are all based upon the principle of a *coercive* treatment being necessary in at least some cases, yet to be used as seldom as possible. In my reply to the queries of the Commissioners, I do not refer to those less direct methods of restraint, but only to the mechanical; and I remarked, "As to the use of persons or mechanical appliances, when physical force is absolutely necessary (and such cases must inevitably occur) my experience is in favour of the latter."

All the means and methods used to regulate the actions of the insane, whether they be simply medicinal, or mechanical, or moral (as seclusion, the cold douche, &c.), may be used with unnecessary frequency, harshness, or cruelty; the objections which apply to one, apply indeed to all. I, for one, therefore, should be glad to see a controversy terminated which has become useless for practical results, and appears not only to be degenerating into personalities, but to present a taint of empiricism. I am, my dear Sir, very truly yours,

York, 13th Nov. 1854. J. Laycock.

